# Recurrent Laryngeal Nerve Injury During Anterior Cervical Discectomy and Fusion (ACDF): A Case Presentation and Review of the Literature

**DOI:** 10.7759/cureus.64603

**Published:** 2024-07-15

**Authors:** Alexandra C Echevarria, Benjamin Hershfeld, Rohit Verma, Margherita Bruni

**Affiliations:** 1 Internal Medicine, Nova Southeastern University Dr. Kiran C. Patel College of Osteopathic Medicine, Davie, USA; 2 Orthopedic Surgery, Northwell Health, Manhasset, USA; 3 Otolaryngology, Northwell Health, Manhasset, USA

**Keywords:** vocal cord paralysis, vocal cord injury, recurrent laryngeal nerve palsy, cervical fusion, anterior cervical discectomy

## Abstract

Anterior cervical discectomy and fusion (ACDF) is a surgical procedure used to manage spine pathology including disc herniation, spondylosis, and myelopathy. During the operation, the vertebral segment of interest is accessed via the anterior neck and the disc space is fully resected along with osteophytes to relieve the compression along the affected nerve. While the procedure is regarded as being highly effective in improving symptoms, there are several complications associated with the surgery that patients should be cautioned about. We present a case of a patient with oropharyngeal and cervical esophageal dysphagia and left vocal cord paralysis following a C5/C6, C6/C7, and C7/T1 ACDF for multilevel cervical stenosis and disc herniation. Otolaryngology evaluation confirmed vocal cord paralysis from recurrent laryngeal nerve palsy (RLNP) and the patient’s symptoms were managed with a vocal cord injection and speech therapy. This report explores the surgical approach for ACDF along with its complications and postoperative care.

## Introduction

Anterior cervical discectomy and fusion (ACDF) is a commonly performed spinal procedure, primarily indicated for cervical radiculopathy, myelopathy, disc herniation, and spinal stenosis [1]. This surgical intervention aims to restore intervertebral disc height, stability, and proper curvature through anterior decompression, interbody grafting, and fusion. During the procedure, the patient is placed in a supine position and the head and neck are secured in a neutral position which is confirmed by fluoroscopy. A prospective incision is made over the vertebral segment of interest and dissection is then performed to part the skin, fascia, and other surrounding structures from the cervical vertebra. During this stage, distraction pins are placed above and below the disc space, and a discectomy is performed followed by decompression of the uncovertebral joints and foramina. Trial implants are then measured and tested in the disc space to determine the correct implant dimensions. Fluoroscopy is again used to confirm the size and placement of the trial before inserting the final implant [2,3]. While ACDF is associated with clinical success, it is not without complications. The complication rate of ACDF procedures is estimated to be between 0.9% and 8.3% with recurrent laryngeal nerve palsy (RLNP) being among the more unusual postoperative issues. The recurrent laryngeal nerve (RLN) is responsible for innervating the intrinsic muscles of the larynx which control the mobility of the vocal cords and allow for ease of speech. RLNP can present as hoarseness immediately after surgery and, in rare cases, as delayed-onset paralysis. The occurrence of RLNP may be attributed to mechanical manipulation during surgery and is diagnosed with direct visualization via laryngoscopy [1,4]. In this case report, we present a case of RLNP following an ACDF procedure and investigate the current literature to inquire about common causes and modes of preventing complications. We discuss the patient's presentation, surgical procedure, and postoperative complications; emphasizing the importance of vigilance in both surgical technique and postoperative monitoring to prevent RLNP. By highlighting the complexities of RLNP and strategies to reduce its occurrence, this case underscores the significance of careful surgical considerations and proactive patient care in ACDF procedures.

## Case presentation

A 51-year-old male with a past medical history of spinal stenosis at C5-C6 and disc herniation at C6-C7 and C7-T1 presented for evaluation of left upper extremity (LUE) paresthesia and pain. The patient endorsed chronic neck and LUE pain which worsened over time and progressed to LUE weakness, numbness, and paresthesia. He pursued conservative treatment options for six months including physical therapy, anti-inflammatory medications, opioids, muscle relaxants, and steroid injections. The patient’s symptoms continued to worsen and led to severe impairment in his activities of daily living secondary to LUE paresthesia, dysesthesia, and significant weakness in the C7 and C8 radicular dermatome distribution.

**Figure 1:**
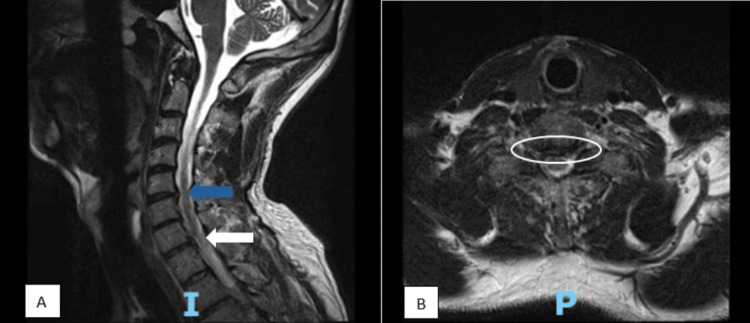
MRI of the cervical spine showing sagittal (A) and axial (B) views. (A) Sagittal view showing multiple disc herniations at C5-C6 (blue arrow) and C7-T1 (white arrow). (B) Axial view of the C5-C6 level revealing narrowing of the spinal cord (white oval).

On physical examination, he was found to have decreased motor strength of the LUE with 3/5 strength in the left deltoid, bicep, and triceps with 5/5 strength in the wrist and hand. He had decreased sensation in the C6, C7, and C8 dermatome distributions. Magnetic resonance imaging (MRI) of the cervical spine demonstrated multilevel disc herniation at C5-C6 and C7-T1 with moderate stenosis of C5-C6 with congenital narrowing of the spinal canal (Figure 1). Due to the patient’s symptom presentation, failure of conservative management, and severity of MRI pathology, he was recommended for surgical intervention.

**Figure 2:**
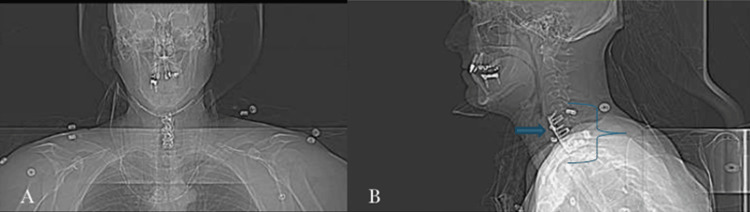
Non-contrast CT scan of the spine showing (A) anterior-posterior and (B) lateral views of the patient with anterior fusion plates spanning C5-T1 (blue arrow) and microscrews anchored within the C5, C6, and T1 vertebral bodies (blue bracket). Intervertebral disc spacers are also seen without osseous fusion across the intervertebral disc spaces.

The patient underwent C5/C6, C6/C7, and C7/T1 ACDF with arthrodesis of the endplates, cage and plate placement, and bone grafting under fluoroscopic guidance. During the procedure, an incision was made over the anterior C6-C7 region through the skin, subcutaneous fat, and overlying platysma muscle. Dissecting scissors were used to dissect the interval between the sternocleidomastoid and the infrahyoid muscles, as well as the prevertebral fascia. Peanut swab dissection was then performed to access the C5-C6, C6-C7, and C7-T1 regions. Discectomy with bony arthrodesis of the endplates, and decompression of the spinal cord and foramina were performed. Subsequently, a 7-mm lordotic Titanium cage was placed in the C5-C6 region. Once bony arthrodesis revealed bleeding cancellous bone, autogenic and allogenic bone grafting material was packed into the cage along with a demineralized bone matrix. The same process was repeated at the subsequent C6-C7 and C7-T1 levels. After the cages were confirmed to be in an excellent position, all distraction pins were removed and a plate measuring 52.5-mm in length was placed from C5-T1 and secured using 14-mm screws. Final fluoroscopic imaging showed the implants to be in the proper position. Postoperative imaging can be seen in Figure 2.

**Figure 3:**
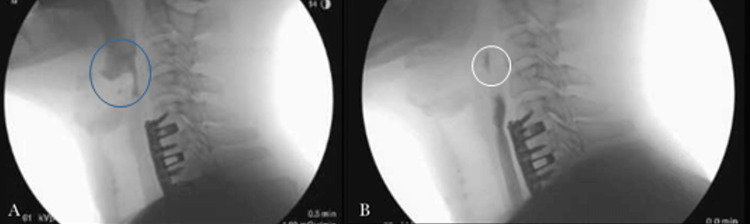
Modified barium swallow study illustrating (A) mild penetration of fluids over the laryngeal surface of the arytenoids and epiglottis (blue circle) and (B) trace to minimal residue at the cervical esophagus post-swallow revealing a delay in clearance of thin liquids in this region (white circle).

Immediately following surgery, the patient noted improved radiculitis and motor strength of the LUE. On postoperative day (POD) 1, the patient had complete resolution of his LUE symptoms but endorsed neck pain, pharyngeal discomfort, and dysphonia. Speech pathology was consulted due to pain with swallowing and voice hoarseness. The patient was diagnosed with oropharyngeal-cervical esophageal dysphagia after a swallow study revealed a delay in the trigger of the swallow reflex, incomplete laryngeal elevation and closure resulting in silent penetration of thin liquids, and post-swallow esophageal residue (Figure 3). He was placed on soft food diet restrictions and observed for improvement in hypophonia and odynophagia. On POD6, the patient underwent direct laryngoscopy and was found to have left unilateral vocal cord paralysis. His left vocal cord was injected with Prolaryn Plus on POD6 for medialization and size augmentation of the paretic cord. Prolaryn Plus is a calcium hydroxylapatite-based gel injection that is administered to the paralyzed vocal fold to manage glottal insufficiency by expanding it to improve speech. He was discharged on POD7 and instructed to follow up with ENT on an outpatient basis. He was subsequently referred to speech therapy for dysphonia, dysphagia, and hoarse, raspy vocal quality. He continues to be followed by ENT and has undergone several laryngoscopies revealing steady improvement of left vocal cord mobility, although it continues to be slightly weaker than the right. While his symptoms have been largely ameliorated with therapy, he continues to experience intermittent voice hoarseness and fatigue.

## Discussion

ACDFs are one of the most commonly performed spinal operations in the US with an average of 137,000 surgeries performed per year [1]. ACDF is a surgical procedure primarily recommended for patients with cervical radiculopathy or myelopathy among a variety of pathologies [5]. This procedure aims to restore the height, stability, and proper curvature of the intervertebral discs through anterior decompression of the disc space [6]. This decompression is followed by the insertion of an interbody graft and fusion. In many cases, additional anterior plating is performed to enhance the stability of the cervical spine, and posterior instrumentation may be used for extra support. During the surgery, an anterior approach is commonly employed to access the cervical spine and facilitate a safe route to visualize and expose the anterior cervical spine through the carotid triangle [7,8]. A discectomy and decompression are performed followed by fusion and fixation to ensure the stability and alignment of the cervical spine [7].

ACDF has been found to have a high rate of clinical success (86.1%) and radicular pain relief (89.3%) [9]. Despite the positive outcomes attributed to ACDF, the intervention is not without drawbacks and complications. Potential complications that should be discussed with patients include worsening myelopathy, RLNP, Horner’s syndrome, respiratory insufficiency, esophageal perforation, and instrument failure. While less common, cases of internal jugular vein thrombosis and phrenic nerve injury have also been reported in the literature [10,11]. Overall morbidity rates for ACDF range from 13.2%-19.3% and the overall mortality rate was found to be 0.1% -1% [1,12].

While RLNP is a rare outcome, it has been extensively reported as a complication of ACDF during retraction or transection of cervical anatomy for appropriate exposure of the spine. RLNP may present with vocal fatigue, dysphonia, or hypophonia, but apart from its effects on vocal quality, it is of particular concern because vocal fold immobility may lead to aspiration from supraglottic laryngeal and pharyngeal dysfunction. Reduced airway protection in such cases is linked to poor laryngeal elevation during swallowing, bolus retention, and respiratory distress [13]. Several therapies have been developed to mitigate the risk of aspiration from glottal insufficiency including injection of materials such as fat, collagen, or hyaluronic acid gels to facilitate vocal fold augmentation and medialization. Injections, such as the one used in the patient presented, not only shift the paretic cord medially but in doing so reinstate the vibro-tactile stimulation from the mobile cord through physical contact [14]. This can function to improve vocal quality and decrease the risk of aspiration.

The anatomic course of the RLN makes it susceptible to injury during neck surgery. The RLN originates from the vagus nerve and has a varying course on the left and right sides of the neck. On the left, the nerve courses below the aortic arch and ligamentum arteriosum, and on the right, the nerve crosses the subclavian artery. On both sides of the neck, however, the nerves course along the tracheoesophageal grooves before entering the larynx. In general, the right RLN tends to be situated in a more anterolateral position and has a greater number of branching extra laryngeal vessels, which theoretically may place it at increased risk of injury during exposure compared to the more medially positioned left RLN [15]. However, several studies published in the literature have investigated the effect of laterality on the risk of RLNP and have found no significant difference between left and right approaches [16,17]. Furthermore, when operating at the lower cervical levels, the risk of RLNP is enhanced by its close proximity to the omohyoid muscle which is dissected and retracted during exposure for proper visualization. This can, in part, explain why nerve injury at the C6-C7 levels is more common than in the upper vertebral levels [8].

RLNP typically emerges secondary to neurapraxia attributed to intraoperative factors such as nerve compression, direct injury from instrumentation, traction, and cuff inflation. Patients with RLNP typically present with hoarseness immediately after surgery due to unilateral vocal cord paralysis but late-onset RLNP may infrequently occur. This progressive development of RLNP may lead to more severe consequences, including permanent vocal cord paralysis or respiratory failure [18]. The incidence of RLNP ranges from 0.9-9% and has not been found to be associated with patient age, body mass index, duration of surgery, or number of levels operated on P > 0.05 [1,19]. Based on this lack of association, the occurrence of postoperative RLNP may depend more on direct mechanical manipulation during surgery, rather than on specific patient or surgical characteristics [19]. Furthermore, a secondary ACDF procedure yielded an RLNP rate of 14.1%, higher than the primary rate of 0.9% to 9% [1,19,20]. This finding suggests an increased risk of hoarseness and dysphagia following reoperation compared to primary procedures.

Many studies have been conducted to understand how to reduce the rate of RLNP following anterior cervical spine surgery (ACSS). Jung and Schramm found that a left-sided approach was associated with a significantly reduced incidence of postoperative and permanent RLNP [21]. These results echo the aforementioned studies citing the anatomical course and branching pattern of the left RLN as contributing factors for a lower risk of palsy compared to the right RLN. Jung and Schramm also found that decreasing the endotracheal (ET) pressure cuff to less than 20 mmHg was associated with a total RLNP rate of 1.3% compared to 6.5% with normal cuff pressure [21]. Similar results were observed in a more recent study concluding that maintaining an ET tube cuff pressure of less than 20 mmHg decreases the risk of RLNP as well as long-term voice hoarseness [22]. Apfelbaum et al. found that interactions by the cervical retraction system with the ET-laryngeal wall displaced the larynx against the shaft of the ET tube, allowing impingement on the vulnerable intralaryngeal segment of the recurrent laryngeal nerve. Monitoring of the ET cuff pressure and release of pressure after retractor replacement or repositioning allowed the ET to recenter within the larynx, leading to decreased rates of RLNP after ACSS [23]. The use of intraoperative nerve monitoring can also be an asset in promptly diagnosing and addressing RNLP, as electromyography (EMG) changes can be tracked in real time. In a recent study by Niljianskul et al., rates of RLNP in patients undergoing ACDF with and without nerve monitoring were evaluated. In the intervention group, an EMG-ET tube was positioned so that the electrodes were at the level of the vocal cords. A signal decrease or loss would prompt the surgeon to reevaluate surgical events and adjust instrumentation accordingly. Results showed there to be higher rates of RLNP in the non-EMG group, but the difference was not statistically significant [24].

## Conclusions

The patient presented in this report had voice hoarseness, dysphonia, and dysphagia after surgery which have improved over time but continue to affect his daily life. The injury was secondary to neuropraxia, nerve contusion, compression, or inflammation of tissue caused by the ET tube and retraction. Preventing RLNP and other complications following ACDF is of paramount importance for spine surgeons. Careful tissue dissection, minimization of tissue traction, and adequate exposure of the surgical site are all essential elements in preventing nerve injury. Proper patient positioning and ET tube management, including cuff pressure monitoring can help reduce the risk of nerve compression or stretching. Post-surgically, a vocal cord function assessment is key to detecting early signs of RLNP and enabling prompt intervention if needed. Furthermore, clear communication with patients about potential risks and complications fosters informed consent and open dialogue. In cases where RLNP does occur, rehabilitation and vocal therapy can play vital roles in helping patients regain vocal cord function and improve their quality of life.
